# Impact of p53 status on TRAIL-mediated apoptotic and non-apoptotic signaling in cancer cells

**DOI:** 10.1371/journal.pone.0214847

**Published:** 2019-04-04

**Authors:** Anna Willms, Hella Schittek, Sascha Rahn, Justyna Sosna, Ufuk Mert, Dieter Adam, Anna Trauzold

**Affiliations:** 1 Division of Molecular Oncology, Institute for Experimental Cancer Research, CCC-North, University of Kiel, Kiel, Germany; 2 Institute of Immunology, University of Kiel, Kiel, Germany; 3 Clinic for General Surgery, Visceral, Thoracic, Transplantation and Pediatric Surgery, University Hospital Schleswig-Holstein, Kiel, Germany; Heinrich-Heine-Universitat Dusseldorf, GERMANY

## Abstract

Due to their ability to preferentially induce cell death in tumor cells, while sparing healthy cells, TNF-related apoptosis-inducing ligand (TRAIL) and agonistic anti-TRAIL-R1 or anti-TRAIL-R2-specific antibodies are under clinical investigations for cancer-treatment. However, TRAIL-Rs may also induce signaling pathways, which result in malignant progression. TRAIL receptors are transcriptionally upregulated via wild-type p53 following radio- or chemotherapy. Nevertheless, the impact of p53 status on the expression and signaling of TRAIL-Rs is not fully understood. Therefore, we analyzed side by side apoptotic and non-apoptotic signaling induced by TRAIL or the agonistic TRAIL-R-specific antibodies Mapatumumab (anti-TRAIL-R1) and Lexatumumab (anti-TRAIL-R2) in the two isogenic colon carcinoma cell lines HCT116 p53^+/+^ and p53^-/-^. We found that HCT116 p53^+/+^ cells were significantly more sensitive to TRAIL-R-triggering than p53^-/-^ cells. Similarly, A549 lung cancer cells expressing wild-type p53 were more sensitive to TRAIL-R-mediated cell death than their derivatives with knockdown of p53. Our data demonstrate that the contribution of p53 in regulating TRAIL-R-induced apoptosis does not correlate to the levels of TRAIL-Rs at the plasma membrane, but rather to p53-mediated upregulation of Bax, favouring the mitochondrial amplification loop. Consistently, stronger caspase-9 and caspase-3 activation as well as PARP-cleavage was observed following TRAIL-R-triggering in HCT116 p53^+/+^ compared to HCT116 p53^-/-^ cells. Interestingly, HCT116 p53^+/+^ cells showed also a more potent activation of non-canonical TRAIL-R-induced signal transduction pathways like JNK, p38 and ERK1/ERK2 than p53^-/-^ cells. Likewise, these cells induced IL-8 expression in response to TRAIL, Mapatumumab or Lexatumumab significantly stronger than p53^-/-^ cells. We obtained similar results in A549 cells with or without p53-knockdown and in the two isogenic colon cancer cell lines RKO p53^+/+^ and p53^-/-^. In both cellular systems, we could clearly demonstrate the potentiating effects of p53 on TRAIL-R-mediated IL-8 induction. In conclusion, we found that wild-type p53 increases TRAIL-R-mediated apoptosis but simultaneously augments non-apoptotic signaling.

## Introduction

TRAIL (TNF-related apoptosis-inducing ligand) binds to four plasma membrane-bound receptors (TRAIL-R1-4). Two of them, TRAIL-R1 and TRAIL-R2, are capable of inducing apoptosis via their intracellular death domain (DD) and are therefore called death receptors. The two other receptors, TRAIL-R3 and TRAIL-R4, lack a functional DD thus are not able to induce cell death. TRAIL-R3, anchored in the plasma membrane via glycosylphosphatidylinositol, contains neither a transmembrane nor a cytoplasmic domain. Thus, this receptor cannot transmit TRAIL-induced signaling. The cytoplasmic domain of TRAIL-R4 is able to induce several non-apoptotic signal transduction pathways but possesses a truncated, non-functional DD. Both TRAIL-R3 and TRAIL-R4 were proposed to negatively regulate TRAIL-induced apoptosis via direct interaction and/or ligand competition with the pro-apoptotic receptors TRAIL-R1/R2 [1–3].

Upon TRAIL ligation, TRAIL death receptors assemble at their intracellular DD the death-inducing-signaling-complex (DISC) composed of FAS-associated protein with death domain (FADD) and pro-caspase-8/10 [4]. Proximity-induced self-cleavage of pro-caspases leads to their activation and dissociation from the multiprotein-complex. In so-called type I cells, efficient DISC formation allows a direct activation of the effector caspases for activating the apoptotic cascade. In contrast, in so-called type II cells, induction of apoptosis by TRAIL requires the intrinsic apoptotic pathway, which includes a mitochondrial activation loop to enhance the initial apoptotic signal. In these cells, activated caspase-8/-10 cleaves Bid to its truncated form tBid, which in turn leads to Bax/Bak-mediated mitochondrial outer membrane permeabilization (MOMP) and release of pro-apoptotic mitochondrial proteins like cytochrome c [5]. In the cytosol, cytochrome c, the apoptotic protease-activating factor 1 (APAF-1) and pro-caspase-9 form an apoptosome, a platform for activation of caspase-9, which in turn is able to activate effector caspases to trigger apoptosis.

TRAIL´s ability to preferentially kill tumor cells, while sparing healthy cells, makes it a promising weapon for targeted tumor therapy [6,7]. However, in addition to induction of cell death, TRAIL-R1/R2 also induce various non-canonical signal transduction pathways, like NF-*κ*B, MAP kinases, AKT, PKC and Src, which may lead, particularly in apoptosis-resistant cells, to increased proliferation, invasion and metastasis [8–13].

TRAIL receptors are known transcriptional targets of the tumor suppressor protein p53, and chemotherapeutic agents potentiate cell death in wild-type p53-expressing tumor cells by enhancing TRAIL-R1/R2 expression at the plasma membrane [14,15]. Therefore, numerous studies described targeting of p53 as a therapeutic strategy to sensitize cancer cells to TRAIL-induced apoptosis [15]. P53, a transcription factor with tumor suppressor function, can be activated by diverse forms of cellular stress like DNA damage, hypoxia, lack of nutrients and cell cycle abnormalities. P53 is one of the most important targets in anti-tumor therapy and most conventional chemotherapeutic agents, as wells as radiation, activate p53 [16]. Depending on the level of the damage, activated p53 triggers either a transient cell cycle arrest, apoptotic cell death or permanent cell cycle arrest (cellular senescence). For example, the intrinsic apoptotic pathway, which senses cellular damage and misbalanced intracellular homeostasis, depends on p53.

In the majority of human cancers, p53 is either mutated or inactivated via complexing with inhibitory proteins like Mdm-2. For such tumors, induction of TRAIL-R-mediated cell death, as a p53-independent pathway, may represent a promising alternative therapeutic strategy. There are several available agonists of TRAIL-Rs, including recombinant human TRAIL or agonistic TRAIL-R-specific antibodies [17]. However, in clinical trials, none of these TRAIL-R agonists led to a therapeutic benefit for cancer patients [18,19]. Although the influence of p53 activation in the course of chemo- and radiotherapy on death-receptor-mediated apoptotic signaling has extensively been studied [15], the sole impact of p53 status on TRAIL-R-induced signaling, in particular on non-apoptotic signaling pathways is still not understood.

In the present study, we investigated side by side the impact of wild-type p53 on apoptotic and non-apoptotic TRAIL-R signaling following treatment with TRAIL or agonistic, receptor-specific antibodies.

## Material and methods

### Cell culture and stimulation

The human colon cancer cell lines HCT116 p53^+/+^ (WT) and HCT116 p53^-/-^ (KO) were kindly provided by Bert Vogelstein and described previously [20]. The colon cancer cell lines RKO p53^+/+^ and RKO p53^-/-^ were purchased from Horizon Discovery, Cambridge, UK. HCT116, RKO, and lung cancer A549 cells all were cultured in RPMI 1640 medium supplemented with 10% FCS, 2 mM glutamine and 1 mM sodium pyruvate. To the cell culture medium for HCT116 p53^-/-^ cells, additionally Geneticin (400 μg/mL) and Hygromycin B (100 μg/mL) were added. The antibiotics were omitted when the cells were seeded for subsequent stimulations. Cells were stimulated with recombinant human TRAIL (100 ng/ml; PeproTech, Hamburg, Germany) or with the agonistic anti-TRAIL-R1- or anti-TRAIL-R2 antibodies Mapatumumab and Lexatumumab (10 μg/ml; Human Genome Sciences, Rockville, USA), respectively. To inhibit caspases, zVAD-fmk (Bachem, Bubendorf, Switzerland; conc. 20 μM) was added to the cell culture medium 1 h prior to the treatment with TRAIL-R-targeting agents. To knock down the expression of p53 in A549 cells, cells were transfected with ON-TARGET plus TP53 or control siRNA (Horizon Discovery, Cambridge, UK) using a Lipofectamine^TM^ RNAiMAX transfection reagent (Invitrogen, California, USA) for 72 h.

### Cell viability assay

HCT116 (p53^+/+^ / p53^-/-^) and RKO (p53^+/+^ / p53^-/-^) cells were seeded (2x10^4^/ well) in 96-well plates grown for 24 h and stimulated with TRAIL, agonistic anti-TRAIL-R1- or anti-TRAIL-R2 antibodies and zVAD-fmk for 24 h. Cell viability was assayed by crystal violet staining as described previously [12].

### Measurement of plasma membrane integrity

HCT116 (p53^+/+^ / p53^-/-^) cells were seeded in 6-Well plates (2x10^5^/ well) grown for 24 h and stimulated with TRAIL or agonistic anti-TRAIL-R1- or anti-TRAIL-R2 antibodies for 24h. Cell death was evaluated by staining of cells with 2 μg/mL of propidium iodide (PI) followed by determination of membrane integrity via flow cytometry by FACSCalibur (Becton Dickinson, Heidelberg, Germany). A population size of 30.000 cells was regarded as representative for data evaluation using FlowJo v10 (FlowJo, LCC, Oregon, US).

### DNA-fragmentation assay

HCT116 (p53^+/+^ / p53^-/-^) and A549 (Ctrl-si / p53-si) cells were seeded in 6-Well plates (2x10^5^/ well) grown for 24 h and stimulated with TRAIL or agonistic anti-TRAIL-R1- or anti-TRAIL-R2 antibodies for 12 h (HCT116 p53^+/+^ / p53^-/-^) or 24 h (A549 Ctrl-si / p53-si). DNA fragmentation was measured using propidium iodide staining followed by flow cytometry analysis as described previously [21]. Briefly, cells and supernatants were collected (10 min, 300 x g, 4°C), washed with PBS and fixed in 50% Ethanol/ PBS for 30 min at 4°C. Cells were treated with 14 μg/ml RNase (Sigma Aldrich, Traufkirchen, Germany) for 30 min at RT and stained with 250 μg/ml PI/ PBS afterwards. Samples were measured within 1h using a FACSCalibur (Becton Dickinson, Heidelberg, Germany). A population size of 30.000 cells was regarded as representative for data evaluation using FlowJo v10 (FlowJo, LCC, Oregon, US).

### Flow cytometric analyses of cell surface expression of TRAIL receptors

Cell surface expression levels of TRAIL receptors were analyzed by flow cytometry. Briefly, cells were detached from culture dishes by treatment with Accutase (Merck, Millipore, Darmstadt, Germany). Afterwards, cells were washed with cold MACS buffer (PBS supplemented with 2% fetal calf serum and 1 mM EDTA) and FcR-blocking was performed with human FcR blocking reagent (Miltenyi Biotec GmbH, Bergisch-Galdbach, Germany) according to the manufacturer’s instructions. For single stainings of TRAIL receptors, 2 x 10^5^ cells were incubated for 30 min at 4°C with the following APC-conjugated antibodies: anti-human TRAIL-R1 (clone #69036; 10 μg/ml), anti-human TRAIL-R2 (clone #71908; 10 μg/ml), anti-human TRAIL-R3 (clone #90906; 10 μg/ml) or anti-human TRAIL-R4 (clone #104918; 10 μg/ml), all purchased from R&D Systems GmbH, Wiesbaden, Germany. Respective isotype control stainings were performed with APC-conjugated mouse IgG_1_ (clone #11711) and mouse IgG_2B_ (clone #13303) antibodies (both from R&D Systems GmbH). Finally, cells were washed twice in cold MACS buffer, resuspended in cold MACS buffer supplemented with 1% PFA and measured within 24 h using a FACSCalibur (Becton Dickinson, Heidelberg, Germany). A population size of 30,000 cells was regarded as representative for data evaluation using FlowJo v10 (FlowJo, LCC, Oregon, US).

### Biotinylation of cell surface proteins

4 x 10^6^ cells were seeded on 150 mm plates and cultured for 36 h. After removal of the medium, cells were washed twice with ice-cold PBS and incubated with 10 ml Sulfo-NHS-SS-Biotin (0.24 mg/ml in PBS) while being gently shaken on ice for 15 min. Then, the cells were washed with ice cold PBS once and incubated for 10 min with 10 ml quenching solution (0.1 mM CaCl_2_, 1 mM MgCl_2_, 100 mM glycine). Thereafter, the cells were washed with ice cold PBS and lysed with RIPA buffer supplemented with Complete Protease Inhibitor Cocktail and PhosphoStop (both from Roche, Mannheim, Germany). Biotinylated proteins were isolated from 400 μg of whole cell lysates using streptavidin conjugated magnetic beads (Thermo Fisher Scientific, Waltham, USA) overnight on a roller at 4°C. The beads were washed three times with TBST, resuspended in 25 μl 2 x Laemmli buffer and boiled for 7 minutes. Finally, buffer containing precipitated proteins was magnetically separated from the beads and 5 μl were loaded onto SDS gels.

### Western blot analysis

Cells were lysed in RIPA buffer supplemented with Complete Protease Inhibitor Cocktail and PhosphoStop (both from Roche, Mannheim, Germany) and Western blot analyses were performed as described previously [22]. Primary antibodies were purchased from: Cell Signaling, Frankfurt, Germany (anti-ERK1/2 (9102), anti-phospho-ERK1/2 (9106), anti-JNK (9252), anti-phospho-JNK (9251), anti-p38 (9217), anti-phospho-p38 (9211), anti-caspase-8 (9746S), anti-caspase-3 (9668), anti-caspase-9 (9502), anti-decoy receptor 2 (8049) and anti-PARP-1 (9542)); Santa Cruz Biotechnology, Heidelberg, Germany (anti-p53 DO-1 (sc-126)); BD Transduction Laboratories, Heidelberg, Germany (anti-Bax (610982) and anti-APAF1 (A92820)); Merck Millipore, Darmstadt, Germany (anti-DR4 (AB16955)); ProScience Incorporated, USA (anti-TRAIL-R2 (2019)); R&D Systems, Wiesbaden, Germany (anti-BID (AF846)); Abcam, Cambridge, UK (anti-TRAIL-3 (AB133658)) and from Sigma-Aldrich (anti-β-actin (A5441)).

### Real-time polymerase chain reaction (RT-PCR)

Cells were homogenized with QIAshredder (Qiagen, Hilden, Germany) and total RNA was isolated with an RNeasy Mini Kit (Qiagen, Hilden, Germany). The expression of IL-8 was studied by RT-PCR using TaqMan assays (Thermo Fisher Scientific, Waltham, USA) and a 7900HT Fast RT-PCR system (Thermo Fisher Scientific). The expression levels were calculated relative to the expression of the housekeeping gene TATA-binding protein (TBP) by ΔΔCT method. Primers were purchased from Thermo Fisher Scientific (TBP (Hs00427620_m1) and IL8 (Hs00174103_m1)).

### Enzyme-linked immunosorbent assay (ELISA)

The IL-8 levels were determined in cell culture supernatants via ELISA (Bio-Techne, Wiesbaden, Germany) according to the manufacturer’s protocol.

### Statistical analysis

Statistical analyses were performed using SigmaPlot v12.5 (Systat, Erkrath, Germany). First, data were tested for normality and equal variance by Shapiro-Wilk and Equal Variance test, respectively. For comparison of two-groups comprising parametric distributed datasets, t-test was applied. Two groups of non-parametric datasets were analyzed with Mann-Whitney Rank Sum test. Statistically significant differences between the groups were assumed at p-values < 0.05 and marked with an asterisk (*).

## Results

### Impact of p53 status on plasma membrane levels of TRAIL receptors

**Fig 1:**
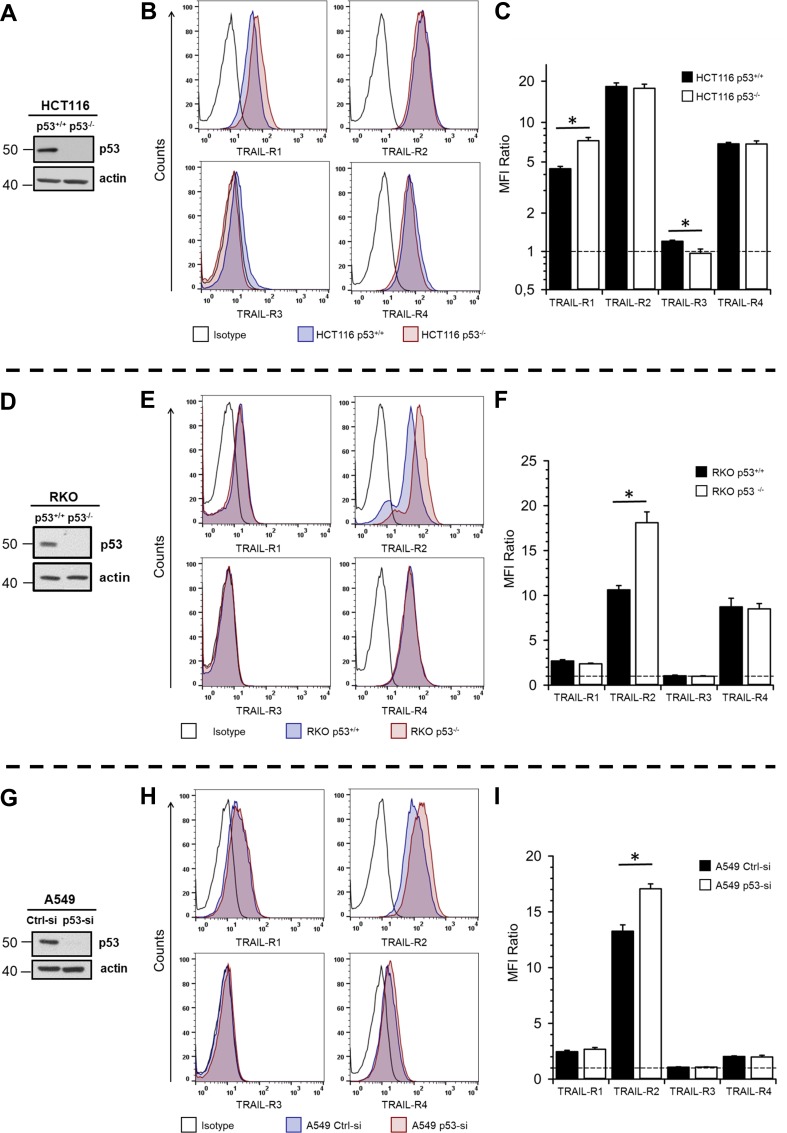
Impact of p53 status on the expression of TRAIL-Rs at the cell surface. The expression of p53 in whole cell lysates of HCT116 p53^+/+^ vs. p53^-/-.^(A), RKO p53^+/+^ vs. p53^-/-.^(D) and A549 cells with and without transient knockdown of p53 (G) was analyzed by Western blot. Detection of β-actin served as control of equal gel loading. For analysis of cell surface expression of TRAIL receptors, HCT116 cells (B, C), RKO cells (E, F) and A549 cells (H, I) were stained with APC-conjugated receptor-specific antibodies and the stainings were measured by flow cytometry. Corresponding APC-conjugated isotype controls were used to validate staining specificity. Shown are representative histograms for TRAIL receptor stainings in HCT116 (B), RKO (E) and A549 (H) cells. Bar charts show mean values ± standard deviation of median fluorescence intensity ratios (TRAIL-R/Isotype) from three independent experiments of TRAIL receptor stainings (C, F, I). * p < 0.05.

Since TRAIL receptors are known transcriptional targets of p53, we first asked whether the p53 status might affect their expression in HCT116 cells. For this purpose, we compared the cell surface levels of TRAIL-R1/R2/R3/R4 in HCT116 p53^+/+^ and p53^−/−^ cells by flow cytometric analyses. As shown in Fig 1A–1C, the plasma membrane levels of TRAIL-R1 were significantly increased in p53^−/−^ cells compared to p53^+/+^ cells. In contrast, the expression of TRAIL-R3, albeit much less pronounced, was higher at the cell surface of p53^+/+^ cells than p53^−/−^ cells. The surface expression levels of TRAIL-R2 and TRAIL-R4 remained unchanged. To validate these data by an independent approach, we biotinylated cell surface proteins and subsequently analyzed the levels of biotinylated TRAIL-Rs in whole cell lysates by Western blotting. The results shown in S1 Fig clearly demonstrate the increased levels of TRAIL-R1 at the cell surface of HCT116 p53^-/-^ when compared to HCT116 p53^+/+^ cells and show that the levels of TRAIL-R3 are slightly downregulated.

These data suggest that the p53 status differentially affects the protein level of TRAIL-R1 and TRAIL-R3 at the cell surface of HCT116 cells. Thus, the absence of p53 increases the abundance of TRAIL-R1, while decreasing the levels of TRAIL-R3. To prove whether this holds true for other cell lines differing in p53 status, we analyzed the expression of TRAIL receptors in another two isogenic colon carcinoma cell lines RKO p53^+/+^ and p53^−/−^ and in addition in a lung cancer cell line A549 with and without transient knockdown of p53. Flow cytometric analyses of TRAIL-R expression at the plasma membrane revealed that in these cell lines, loss of p53 (RKO cells; Fig 1D–1F) or its knockdown (A549 cells; Fig 1G–1I) resulted in a significant up-regulation of TRAIL-R2 at the cell surface. Again, biotinylation experiments performed with RKO cells confirmed these results (S1B Fig).

Summing up, we show that wild-type p53 decreases the plasma membrane expression of TRAIL death receptors in a cell line-dependent manner. Thus, in HCT116 cells the presence of p53 decreases the levels of TRAIL-R1- whereas in A549 and RKO cells TRAIL-R2 is affected.

### Impact of p53 status on apoptotic TRAIL-R signaling

**Fig 2:**
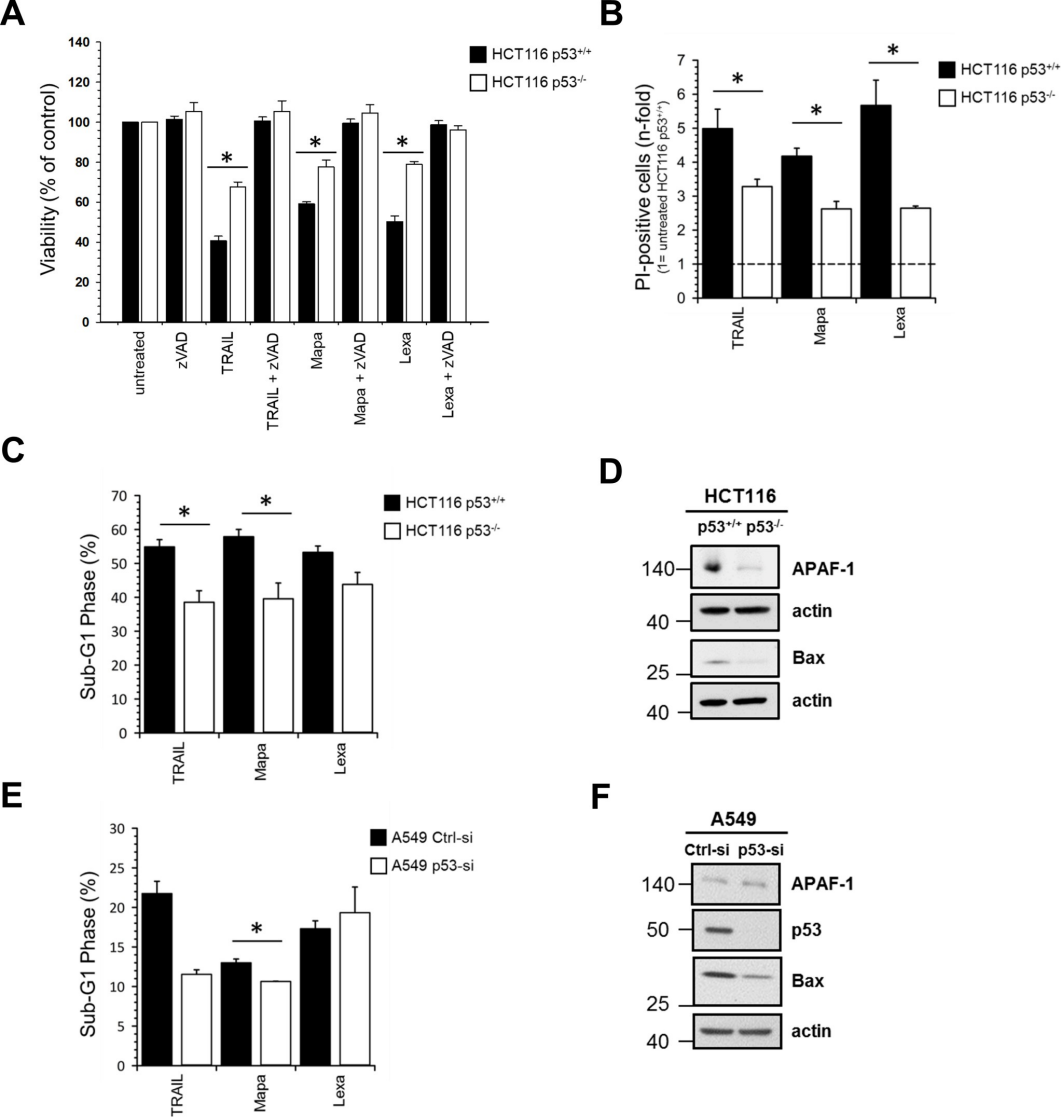
Inhibition of p53 expression decreases TRAIL receptor-induced apoptosis. HCT116 p53^+/+^ and HCT116 p53^-/-^ cells were stimulated either with TRAIL (100 ng/ml), Mapatumumab (10 μg/ml) or Lexatumumab (10 μg/ml) for 24 h with or without zVAD-fmk. Cell viability was determined by crystal violet staining (A) or by PI-staining followed by flow cytometry (B). In addition, DNA-fragmentation assay was performed with HCT116 p53^+/+^ and HCT116 p53^-/-^ (C) and A549 cells with and without transient knockdown of p53 (E). Results are shown ± SD of three biological replicates (n = 3) (A-C) or ± SD of two to three biological replicates (n = 2–3) (E). * p<0.05. Impact of p53 expression on the cellular levels of APAF-1 and Bax was analyzed in whole cell lysates of HCT116 cells (D) and A549 (F) by Western blot. The level of actin was determined in parallel and served as loading control.

In order to investigate whether the p53 status affects TRAIL-R-mediated cell death, we treated HCT116 p53^+/+^ and HCT116 p53^-/-^ cells with TRAIL or with agonistic antibodies against TRAIL-R1 (Mapatumumab) or TRAIL-R2 (Lexatumumab) and determined cell viability by crystal violet staining (Fig 2A), propidium iodide (PI)-staining followed by flow cytometry (Fig 2B) and by DNA-fragmentation assay (Fig 2C). We found that both cell lines were sensitive to TRAIL receptor triggering and that cell death was dependent on activation of caspases since it could be completely inhibited by pre-treatment with the pan-caspase inhibitor zVAD-fmk. Importantly, all assays revealed that, irrespective of the treatment, p53^+/+^ cells were significantly more sensitive towards TRAIL-R-mediated cell death than the p53^-/-^ cells. Similarly, knockdown of p53 in A549 cells significantly reduced TRAIL- and Mapatumumab-induced cell death (Fig 2E), whereas RKO p53^+/+^ and p53^−/−^ were apoptosis-resistant, irrespective of p53 status (S2 Fig).

**Fig 3:**
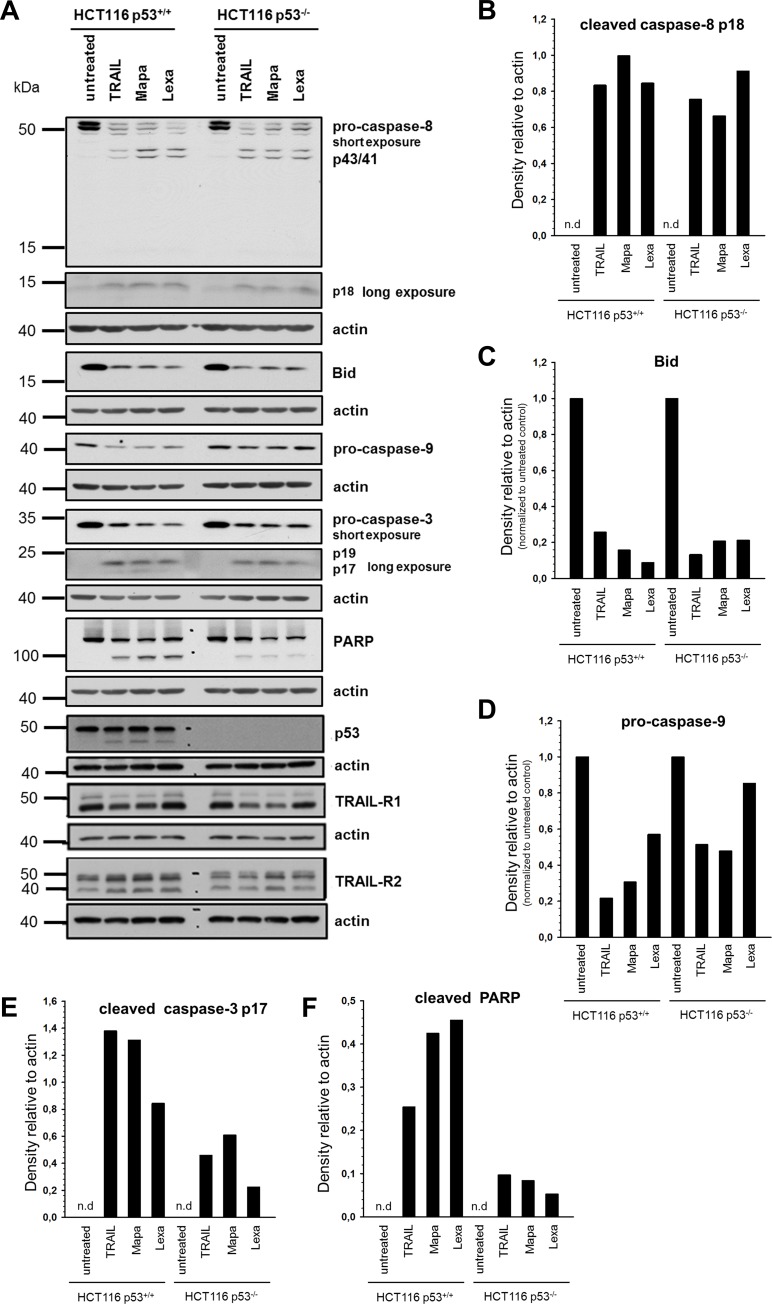
Impact of p53 on TRAIL receptor-induced apoptotic signal transduction pathways in HCT116 cells. HCT116 p53^+/+^ and HCT116 p53^-/-^ cells were stimulated with TRAIL (100 ng/ml), Mapatumumab (10 μg/ml) or Lexatumumab (10 μg/ml) for 24 h and the cleavage of pro-caspase-8, Bid, pro-caspase-9, pro-caspase-3 and PARP-1, as markers of different steps of the apoptotic signaling pathway and TRAIL-R1/R2 and p53 were analyzed in whole cell lysates by Western blot (A). Blots are shown for one representative experiment out of three performed. Western blot bands of caspase-8 p18, Bid, pro-caspase-9, caspase-3 p17 and cleaved PARP-1 were analyzed by densitometry (B-F). Intensity of each band was normalized to the respective actin.

Analyses of apoptotic signal transduction pathways following treatment with TRAIL, Mapatumumab or Lexatumumab in HCT116 cells revealed no significant differences in the activation of caspase-8, except for Mapatumumab treatment, between p53^+/+^ and p53^-/-^ cells (Fig 3A and 3B). Since HCT116 cells are type II cells and therefore induction of apoptosis by TRAIL requires a mitochondrial activation loop to enhance the initial apoptotic signal, we further analyzed Bid-cleavage and activation of caspase-9 as markers for activation of the mitochondrial apoptosis pathway [23,24]. Whereas no changes in Bid cleavage could be observed, we found clearly enhanced activation of caspase-9 in p53^+/+^ cells compared to p53^-/-^ cells (Fig 3A, 3C and 3D).

Consequently, conversion of the effector caspase-3 into p17 and p19 active fragments was stronger in p53^+/+^ cells compared to p53^-/-^ cells and the cleavage of its well established target, the DNA repair like poly(ADP-ribose) polymerase 1 (PARP-1), more prominent (Fig 3A, 3E and 3F).

Concerning the total (rather than plasma membrane) levels of TRAIL death receptors in untreated cells, we found that while the expression of TRAIL-R1 remained unchanged between the cell lines, p53^+/+^ cells expressed slightly more TRAIL-R2 than p53 knockout cells. Of note, in both cell lines, stimulation of TRAIL receptors with TRAIL or Mapatumumab resulted in a decrease of TRAIL-R1 (Fig 3A). In contrast, stimulation with TRAIL, Mapatumumab or Lexatumumab resulted in an increase of TRAIL-R2 in p53^+/+^ cells. In p53^-/-^ cells, an increase of TRAIL-R2 was only visible after stimulation with Mapatumumab or Lexatumumab (Fig 3A).

Interestingly, an additional protein band of approximately 45 kDa, could be detected by p53-specific antibodies in treated but not in untreated control cells, suggesting that it might represent a cleavage product of full length p53 (Fig 3A).

Moreover, we examined the expression levels of the p53 targets Bax and APAF-1, which are important pro-apoptotic factors in type II cells. The cellular levels of both proteins were decreased in HCT116 p53^-/-^ cells compared to HCT116 p53^+/+^ cells (Fig 2D). Similarly, downregulation of p53 in A549 cells led to strong decrease in Bax levels. However, in these cells the levels of APAF-1 remained unchanged (Fig 2F).

In summary, these results revealed that p53 is an important positive regulator of TRAIL-R-induced apoptosis in cancer cells.

### Impact of p53 status on non-apoptotic signaling

**Fig 4:**
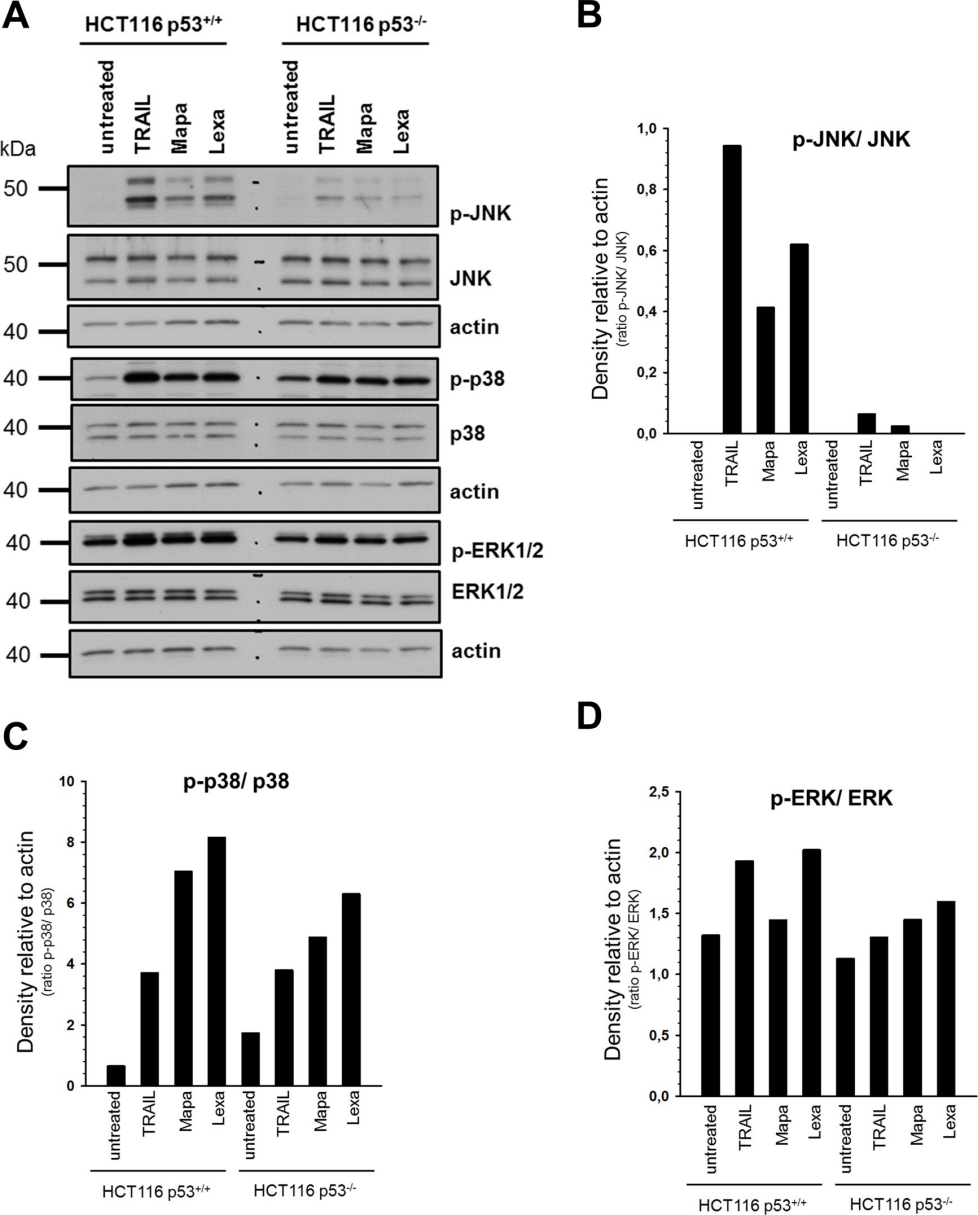
Impact of p53 status on TRAIL-R-mediated non-apoptotic signaling pathways in HCT116 cells. HCT116 p53^+/+^ and HCT116 p53^-/-^ cells were stimulated either with TRAIL (100 ng/ml), Mapatumumab (10 μg/ml) or Lexatumumab (10 μg/ml) for 3 h. (A) Whole cell lysates were analyzed for the phosphorylation/activity status and overall expression of various proteins associated with TRAIL-mediated non-apoptotic signaling pathways by Western blot. The level of actin was determined in parallel and served as loading control. Blots are shown for one representative experiment out of three performed. The ratios of p-JNK/ JNK, p-p38/ p38 and p-ERK1/2/ ERK1/2 were analyzed by densitometry (B-D). Intensity of each band was normalized to the respective actin.

Besides the induction of apoptosis, TRAIL-R1 and TRAIL-R2 are able to induce several non-canonical signal transduction pathways [25]. To investigate whether p53 status influences these cellular responses, we analyzed changes in the phosphorylation/activity of MAP-kinases using phospho-specific antibodies (Fig 4A). As a control, the overall cellular expression levels of JNK, p38 and ERK1/2 were analyzed in parallel. Although the expression levels of the MAP-kinases JNK, ERK1/2 and p38 were similar in both HCT116 p53^+/+^ and p53^-/-^ cells, Western blot analyses revealed that stimulation with TRAIL or TRAIL-R-specific agonistic antibodies induced higher activation-related phosphorylation status of MAP-kinases in p53^+/+^ cells than in p53^-/-^ cells (Fig 4A–4D). Interestingly, in p53^+/+^, but not in p53^-/-^ cells, TRAIL was a stronger inductor of JNK and p38 than agonistic antibodies. Analyses of the activation-related phosphorylation status of MAP-kinases in RKO p53^+/+^ and p53^−/−^ cells by Western Blot revealed similar results (S3A Fig).

**Fig 5:**
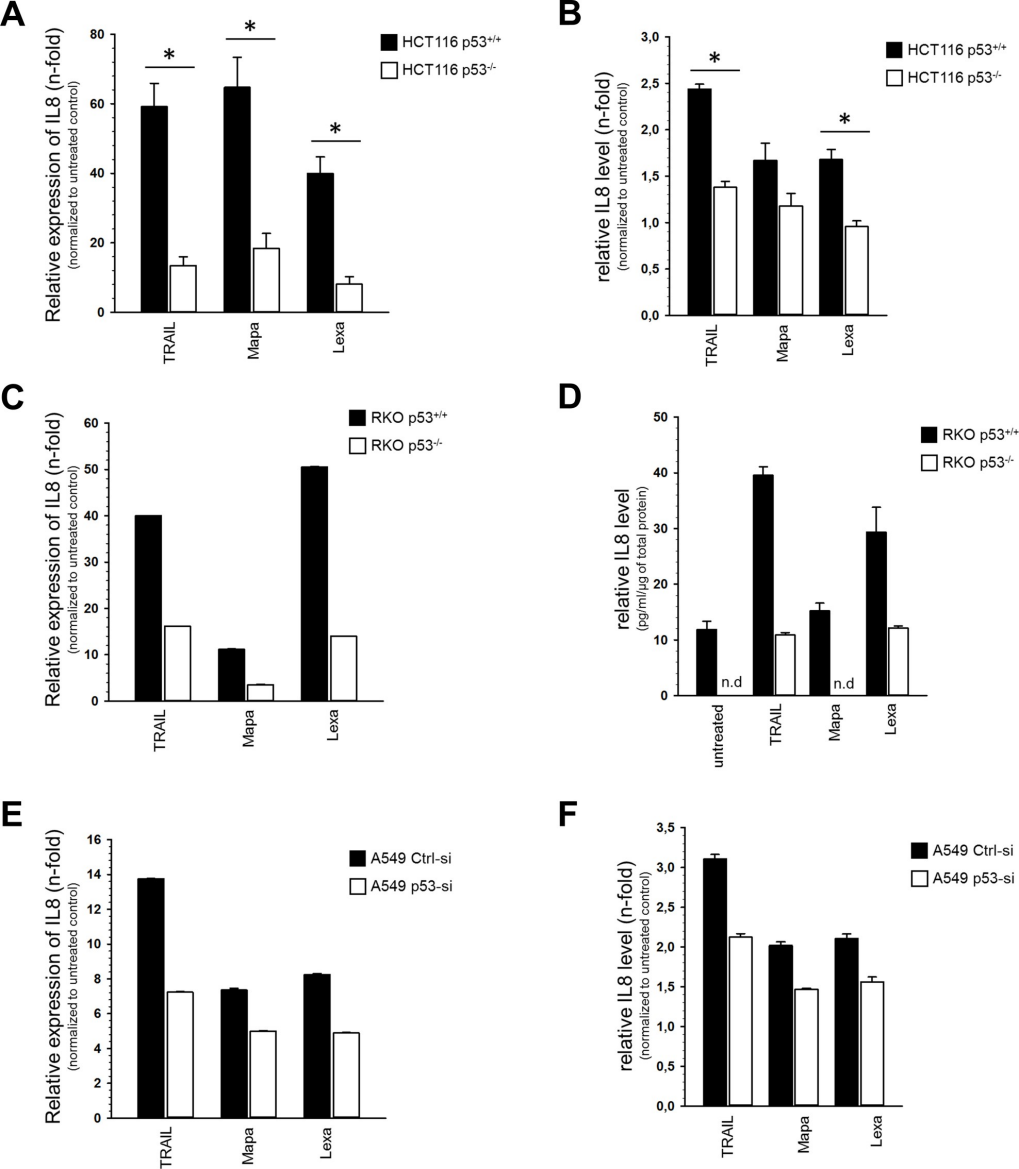
Impact of p53 status on TRAIL-R-mediated IL8 induction. HCT116 cells, RKO cells and A549 cells were stimulated either with TRAIL (100 ng/ml), Mapatumumab (10 μg/ml) or Lexatumumab (10 μg/ml) for 3 h. Relative expression of IL-8 mRNA level (normalized to TBP) was analyzed by qPCR (A, C, E). Relative expression of IL-8 protein level was analyzed by ELISA (B, D, F). Thee independent experiments, each performed in triplicates ± SD (n = 3) are shown in (A, B, D). One representative experiment out of three performed, each performed in triplicates ± SD are shown in (C, E, F).

To determine whether activation of TRAIL receptors might be associated with an acquisition of pro-tumoral cellular properties, we analyzed the expression of pro-inflammatory cytokine IL-8, which has been associated with proliferation, chemotaxis, migration and angiogenesis [26]. Using quantitative real-time PCR and ELISA we found that the expression of IL-8 increased, on both mRNA and protein level following TRAIL receptor stimulation in all cell lines studied (Fig 5). However, these effects were clearly more pronounced in cells expressing p53 than in cells with p53-KO (HCT116 (Fig 5A and 5B), RKO (Fig 5C and 5D)) or p53-KD (A549 (Fig 5E and 5F).

Summing up, these results show that TRAIL, Lexatumumab and Mapatumumab are potent activators of pro-inflammatory signaling in HCT116-, RKO- and A549 cells, especially in the presence of wild-type p53.

## Discussion

Although the interdependencies of TRAIL-R- and p53-functions have long been recognized the consequences thereof, particularly in the case of potential anti-cancer therapies, are still not clear. It has been shown previously, that genes encoding TRAIL-R1-R4, all are transcriptional targets of wild-type p53. Notably, TRAIL-R2 has been identified as a bona fide p53-target induced by most p53-activating agents. Consequently, it has been proposed that drugs activating p53 could sensitize cancer cells to TRAIL-induced apoptosis [15]. Interestingly, however, it seems that the activation of the expression of the particular TRAIL receptor in cells treated with p53-activating agents is cell type- and stimulus-specific. For instance, following treatment with 5-FU or Adriamycin, wild-type p53-expressing HCT116 cells induced the expression of TRAIL-R2, which in turn potentiated p53-induced apoptosis [27]. On the other hand, in the same HCT116 cells, treatment with Oxaliplatin induced the expression of TRAIL-R3 instead of TRAIL-R2 and inhibited TRAIL-mediated apoptosis. In addition to the obviously unpredictable effects of targeting p53 on TRAIL-mediated cell death, the relevance of p53 status for TRAIL-induced apoptosis also seems to be unforeseeable. Thus, using various tumor cell lines and primary xenograft tumor cells, Wachter and colleagues showed that the influence of p53 in its wild-type or mutated conformation on TRAIL-induced cell death depends on the cell type studied and can vary being either positive, negative or irrelevant [28].

In addition to induction of cell death, TRAIL receptors are capable of inducing adverse, malignancy-promoting signal transduction pathways. The eventuality that p53 could also influence these functions has to be taken into account when considering TRAIL-R-triggering as a therapeutic option. In the present study, we therefore set out to determine side by side the effects of p53-status on TRAIL receptor-mediated apoptotic and pro-inflammatory signaling. We treated the cells with recombinant soluble TRAIL as well as with TRAIL receptor-specific drugs Mapatumumab and Lexatumumab, both already tested in clinical trials. With regard to apoptotic signaling, we found that p53^+/+^ HCT116 cells were significantly more sensitive to apoptosis induction than p53^-/-^ HCT116 cells. Similarly, parental, wild-type p53-expressing A549 cells were more sensitive to TRAIL and Mapatumumab than A549 cells in which p53 was knocked down via siRNA treatment.

The higher apoptotic sensitivity of p53^+/+^ HCT116 cells compared to their p53 KO counterparts observed by us has independently been reported by Galligan and coworkers [27], but its reason is unknown. This phenomenon could not be explained by TRAIL-R expression-status since p53^+/+^ cells showed upregulated levels of anti-apoptotic TRAIL-R3 and downregulated levels of death receptor TRAIL-R1 at the cell surface. Thus, our data support previously reported phenomenon that the sensitivity of cells to TRAIL does not necessarily correlate with the levels of TRAIL receptors at the cell surface [29].

Interestingly, the increased level of TRAIL-R1 at the cell surface of HCT116 p53^-/-^ cells cannot be observed when the total amounts of TRAIL-R1 are analyzed by Western blot of whole cell lysates. These results suggest that downregulation of TRAIL-R1 at the surface of HCT116 p53^+/+^ cells compared to their knockout counterparts might reflect the differential trafficking of this receptor in both cell lines. Interestingly, in control A549 and RKO p53^+/+^ cells, TRAIL-R2, instead of TRAIL-R1, was decreased at the cell surface compared to cells with p53-KD or KO, respectively. It is widely accepted that tumor cells have different preferences for TRAIL-R usage following TRAIL-stimulation (for review see [30]). Thus, some cells use TRAIL-R1, others preferentially TRAIL-R2. Furthermore, endogenous, tumor cell-derived TRAIL may lead to a constitutive endocytosis of the death receptors and, consequently, to a lower expression of these receptors at the cell surface as has been demonstrated i.e. for breast cancer cells [31]. It is therefore possible that tumor cells constitutively stimulate themselves and, in dependence on the receptor usage, internalize more TRAIL-R1 or TRAIL-R2. Since p53 is a transcriptional regulator of the *trail* gene, it is possible that wild-type p53 cells produce higher amounts of TRAIL than p53-KO cells, which in consequence leads to a stronger internalization of the particular TRAIL-R (either R1 or R2). Of note, HCT116 cells signal preferentially via TRAIL-R1 [30]. Thus, in these cells, endogenous TRAIL could lead to higher internalization rate of TRAIL-R1 than TRAIL-R2.

The pro-apoptotic role of p53 in TRAIL-mediated cell death might be explained by the fact that several targets of p53 like Bax, APAF1 and Bid are important mediators of TRAIL-induced cell death [32–34]. Accordingly, we observed higher expression levels of Bax and APAF1 in p53^+/+^ cells than in p53^-/-^ cells and of Bax in A549-Ctrl cells compared to A549-p53-KD cells.

Moreover, the basal level of TRAIL-R2, which is also a p53 target, was slightly enhanced in p53^+/+^ cells compared to p53^-/-^ cells. Although analysis of the TRAIL-R2 expression at the plasma membrane showed no differences in both cell lines, intracellular TRAIL-R2 might delocalize from the cytoplasm to the plasma membrane and contribute to stronger apoptosis induction in p53^+/+^ cells. In addition to localization at the plasma membrane, TRAIL-R1 and TRAIL-R2 are often found intracellularly in many cells types, including HCT116 cells [35,36].

Recently, it has been demonstrated that TRAIL and chemotherapeutic drugs may cause the cleavage of p53 into pro-apoptotic fragments via activation of caspase-3. In HCT116 and other p53 wild-type expressing cells, these p53-fragments translocated into the mitochondria leading to the release of cytochrome c and improvement of TRAIL/chemotherapeutics-induced apoptosis [37]. Since we also observed generation of such p53-fragments following TRAIL receptor triggering, this mechanism could also account for the enhanced apoptosis sensitivity of HCT116 p53^+/+^ compared to HCT116 p53^-/-^ cells.

In addition to stronger activation of apoptotic signaling, HCT116 p53^+/+^ cells responded to TRAIL receptor-triggering with stronger induction of pro-inflammatory pathways than p53^-/-^ cells. We observed a similar p53-mediated enhancement of pro-inflammatory IL-8 expression in two other cell lines, RKO and A549, suggesting the more general character of these observations. The capability of TRAIL to induce IL-8 expression has been demonstrated by us and others for different tumor cells [12,38–40]. Importantly, a pro-tumoral role of IL-8 has been established in many cancer entities. Specifically, elevated levels of IL-8 have been linked to enhanced cancer cell proliferation, invasion, metastasis, chemoresistance, neo-angiogenesis and to tumor immunosuppression [41]. Recently, TRAIL-induced pro-inflammatory secretome have been shown to modulate tumor immune microenvironment [40]. The mechanisms how p53 supports TRAIL-R-mediated pro-inflammatory signaling is not known by now.

Our data showing the function of p53 as a factor potentiating the non-apoptotic, pro-inflammatory signaling of TRAIL death receptors are potentially of clinical relevance since it has been reported that 5-FU treatment leads to the induction of TRAIL expression in NK cells in vivo [42]. It is well established that NK cells use TRAIL for the immune surveillance against tumor cells. Intriguingly, a combination of low/medium TRAIL-R1 level and high TRAIL-R3 level in primary colorectal carcinoma cells expressing p53 is associated with a poor response to 5-FU-based chemotherapy and with shorter progression-free survival [43]. This phenomenon might be explained by our data, indicating that p53 promotes the activation of pro-survival and metastatic pathways in response to TRAIL.

In summary, our findings provide that although wild-type p53-expressing cells are more sensitive towards TRAIL-R-mediated cell death than p53-KO or p53-KD cells, TRAIL-R-mediated pro-inflammatory pathways are concomitantly also stronger induced in these cells. Thus, TRAIL-R-targeted anti-cancer therapy in p53^+/+^ expressing cells involves the risk of promoting pro-survival and metastatic pathways in response to TRAIL or TRAIL-R-agonistic antibodies. Whether TRAIL-R-mediated apoptotic or non-apoptotic signaling dominates and which factors influence the switch between these pathways will be the subject of further investigation.

## Supporting information

S1 Fig TRAIL receptor expressions on the cell surface of HCT116 and RKO cells. Extracellular regions of surface proteins on the cell surface of HCT116 p53^+/+^/ p53^-/-^ and RKO p53^+/+^/ p53^-/-^ cells were biotinylated with Sulfo-NHS-SS-Biotin. Cells were lysed with RIPA buffer and biotinylated proteins purified via streptavidin conjugated magnetic beads from equal μg of protein lysates. TRAIL receptor expression was analyzed by western blot of purified proteins using TRAIL-R-specific antibodies. Actin serves as a loading control for biotin pull-down.
(TIF)

S2 Fig RKO p53^+/+^ and p53^-/-^ cells are resistant to TRAIL-R-mediated cell death. RKO p53^+/+^ and RKO p53^-/-^ cells were stimulated either with TRAIL (200 ng/ml), Mapatumumab (10 μg/ml) or Lexatumumab (10 μg/ml) for 24 h with or without zVAD-fmk (20 μM). Cell viability was determined by crystal violet staining (A). Results are shown ± SD of three biological replicates (n = 3).
(TIF)

S3 Fig Impact of p53 status on TRAIL-R-mediated non-apoptotic signaling pathways in RKO cells. RKO p53^+/+^ and RKO p53^-/-^ cells were stimulated either with TRAIL (200 ng/ml), Mapatumumab (10 μg/ml) or Lexatumumab (10 μg/ml) for 3 h. Whole cell lysates were analyzed for the phosphorylation/activity status and overall expression of various proteins associated with TRAIL-mediated non-apoptotic signaling pathways by Western blot (A). Blots are shown for one representative experiment out of three performed.
(TIF)
