# Supplementary figures and images for: Impact of p53 status on TRAIL-mediated apoptotic and non-apoptotic signaling in cancer cells
Source: PLoS One. 2019 Apr 4;14(4):e0214847. doi: 10.1371/journal.pone.0214847 (PMC6448923; doi:10.1371/journal.pone.0214847)

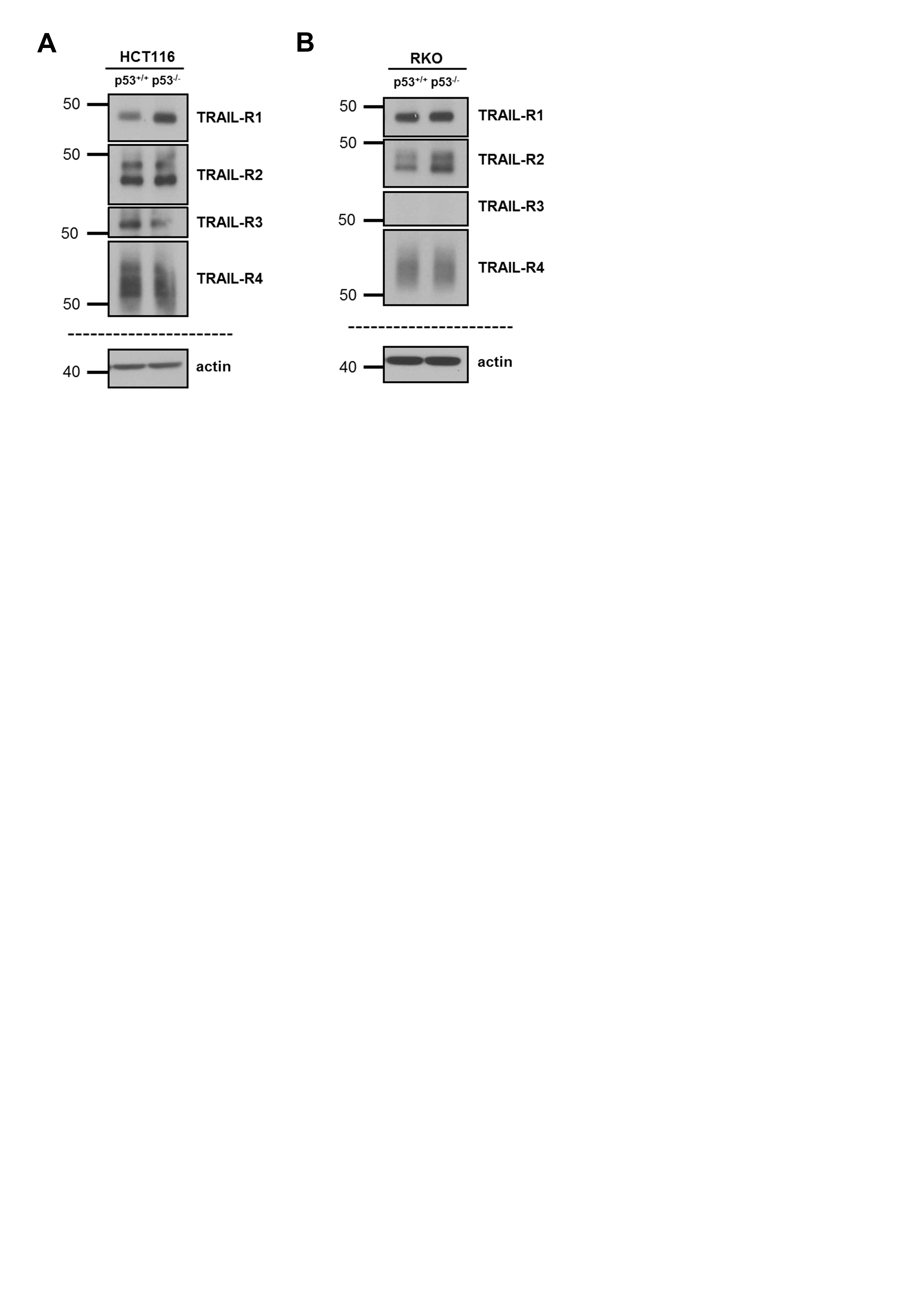

Supplement: S1 Fig — Extracellular regions of surface proteins on the cell surface of HCT116 p53+/+/ p53-/- and RKO p53+/+/ p53-/- cells were biotinylated with Sulfo-NHS-SS-Biotin. Cells were lysed with RIPA buffer and biotinylated proteins purified via streptavidin conjugated magnetic beads from equal μg of protein lysates. TRAIL receptor expression was analyzed by western blot of purified proteins using TRAIL-R-specific antibodies. Actin serves as a loading control for biotin pull-down. (TIF) [file pone.0214847.s001.tif]

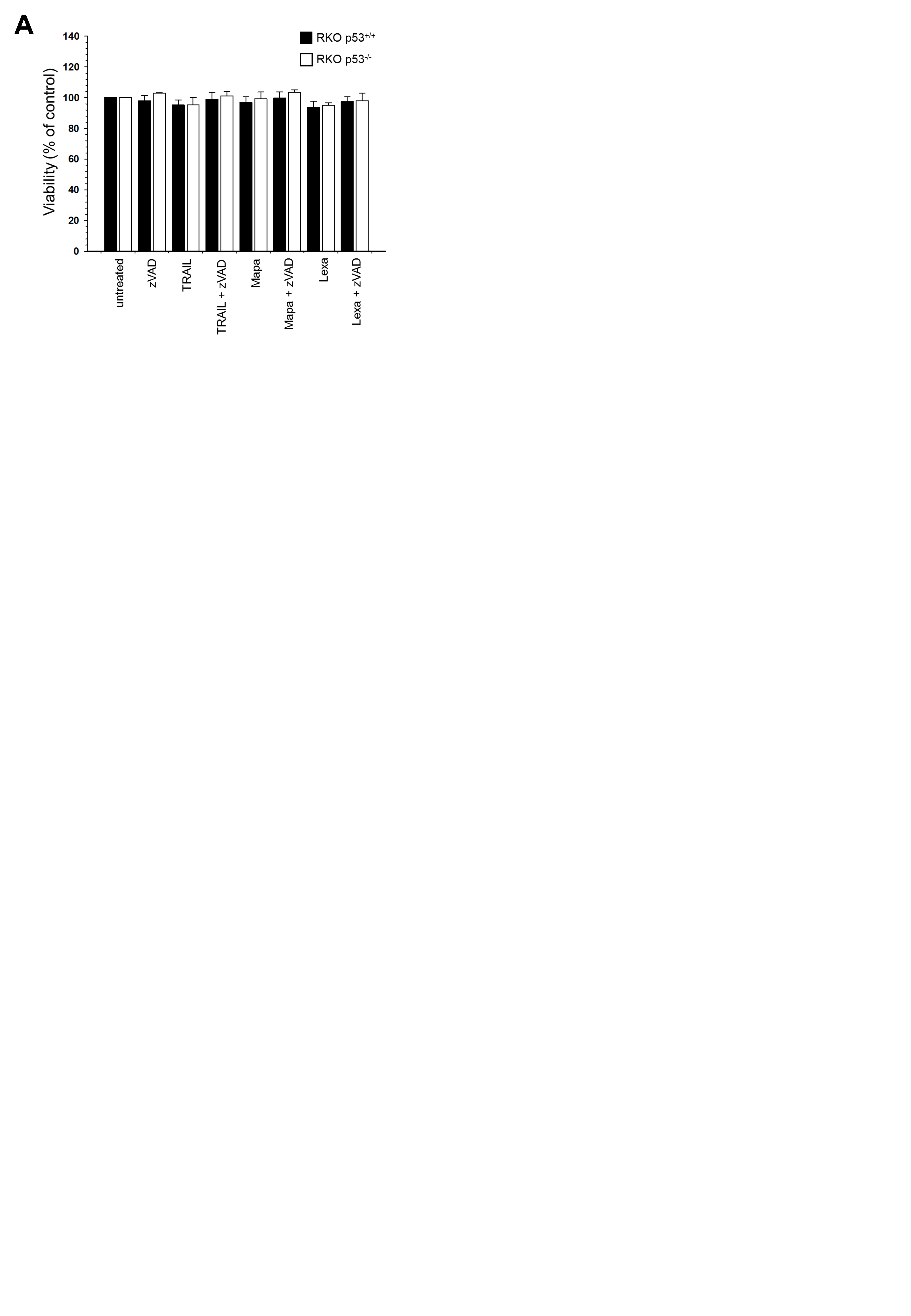

Supplement: S2 Fig — RKO p53+/+ and RKO p53-/- cells were stimulated either with TRAIL (200 ng/ml), Mapatumumab (10 μg/ml) or Lexatumumab (10 μg/ml) for 24 h with or without zVAD-fmk (20 μM). Cell viability was determined by crystal violet staining (A). Results are shown ± SD of three biological replicates (n = 3). (TIF) [file pone.0214847.s002.tif]

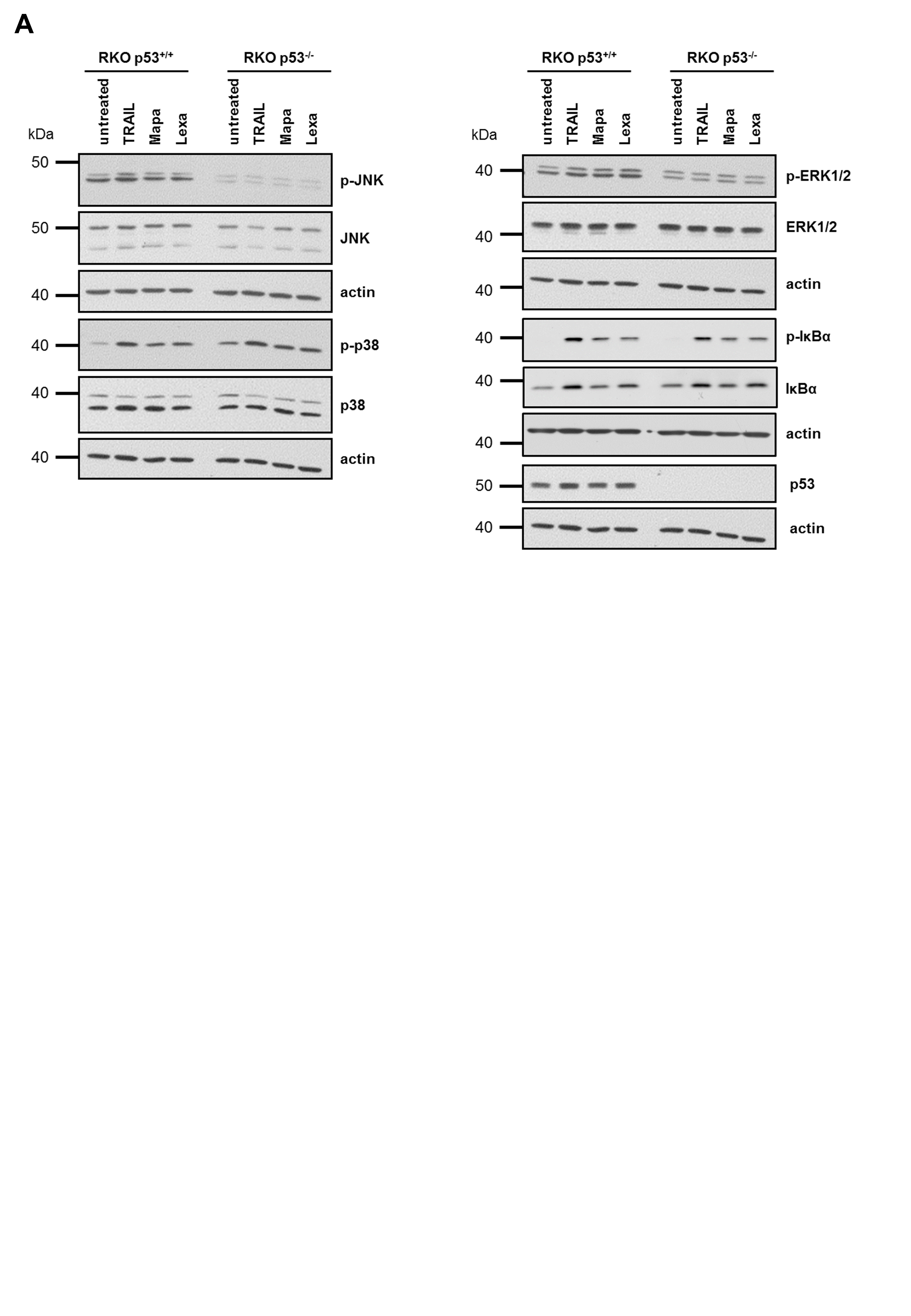

Supplement: S3 Fig — RKO p53+/+ and RKO p53-/- cells were stimulated either with TRAIL (200 ng/ml), Mapatumumab (10 μg/ml) or Lexatumumab (10 μg/ml) for 3 h. Whole cell lysates were analyzed for the phosphorylation/activity status and overall expression of various proteins associated with TRAIL-mediated non-apoptotic signaling pathways by Western blot (A). Blots are shown for one representative experiment out of three performed. (TIF) [file pone.0214847.s003.tif]
